# Early calcineurin-inhibitor to belatacept conversion in steroid-free kidney transplant recipients

**DOI:** 10.3389/fimmu.2022.1096881

**Published:** 2022-12-19

**Authors:** Ibrahim Tawhari, Patrick Hallak, Sofia Bin, Fatmah Yamani, Maria Safar-Boueri, Aazib Irshad, Joseph Leventhal, Mohammed Javeed Ansari, Paolo Cravedi, Lorenzo Gallon

**Affiliations:** ^1^ Department of Medicine, Nephrology, Northwestern University Feinberg School of Medicine, Chicago, IL, United States; ^2^ Department of Medicine, Nephrology, King Khalid University College of Medicine, Abha, Saudi Arabia; ^3^ Department of Medicine, Nephrology, Icahn School of Medicine at Mount Sinai, New York, NY, United States; ^4^ Nephrology, Dialysis and Renal Transplant Unit, IRCCS - Azienda Ospedaliero-Universitaria di Bologna, Alma Mater Studiorum University of Bologna, Bologna, Italy

**Keywords:** belatacept, tacrolimus, mycophenolate mofeti, renal transplant, kidney transplant

## Abstract

**Background:**

Belatacept (Bela) was developed to reduce nephrotoxicity and cardiovascular risk that are associated with the chronic use of Calcineurin inhibitors (CNIs) in kidney transplant recipients. The use of Bela with early steroid withdrawal (ESW) and simultaneous CNI avoidance has not been formally evaluated.

**Methods:**

At 3 months post-transplant, stable kidney transplant recipients with ESW on Tacrolimus (Tac) + mycophenolate (MPA) were randomized 1:1:1 to: 1) Bela+MPA, 2) Bela+low-dose Tac (trough goal <5 ng/mL), or 3) continue Tac+MPA. All patients underwent surveillance graft biopsies at enrollment and then at 12, and 24 months post-transplant. Twenty-seven recipients were included; 9 underwent conversion to Bela+MPA, 8 to Bela+low-dose Tac and 10 continued Tac+MPA. Serial blood samples were collected for immune phenotyping and gene expression analyses.

**Results:**

The Bela+MPA arm was closed early due to high rate of biopsy proven acute rejection (BPAR). The incidence of BPAR was 4/9 in Bela+MPA, 0/8 in Bela+low dose Tac and 2/10 in Tac+MPA, P= 0.087. The Bela+low-dose Tac regimen was associated with +8.8 mL/min/1.73 m^2^ increase in eGFR compared to -0.38 mL/min/1.73 m^2^ in Tac+MPA, P= 0.243. One graft loss occurred in the Bela+MPA group. Immunophenotyping of peripheral blood monocyte count (PBMC) showed that CD28^+^CD4^+^ and CD28^+^CD8^+^ T cells were higher in Bela+MPA patients with acute rejection compared to patients without rejection, although the difference did not reach statistical significance.

**Conclusions:**

Our data indicate that, in steroid free regimens, low-dose Tac maintenance is needed to prevent rejection when patients are converted to Bela, at least when the maneuver is done early after transplant.

## Introduction

Since the development of calcineurin inhibitors (CNIs) in the 1980s, the rate of early acute rejection in kidney transplant recipients has dramatically declined leading to excellent short-term outcomes, but long-term graft survival has increased only slightly (1). This is primarily the consequence of the CNI adverse effects, such as hypertension, dyslipidemia and post-transplant diabetes mellitus, which augment cardiovascular morbidity and mortality even with a functioning graft (2, 3). Furthermore, the exposure to CNIs has been associated with acute and chronic nephrotoxicity. Vasoconstriction of afferent arterioles is the main mechanism by which CNIs cause an acute decrease in glomerular filtration rate (GFR), while arteriolar hyalinosis, glomerulosclerosis, and tubulointerstitial fibrosis are associated with chronic CNI nephrotoxicity (4–6). Therefore, alternative CNI-free immunosuppressive regimens have been proposed to minimize these adverse effects.

Belatacept (Bela) was developed to improve long-term outcomes in kidney transplant patients by avoiding the toxic effects of CNIs. It is a human fusion protein composed of an Fc fragment of IgG1 immunoglobulin linked to the extracellular domain of CTLA-4, which blocks the CD80/CD86 costimulation signals during the interaction between antigen presenting cells and T cells, preventing their activation. It was shown to be noninferior to CNIs and associated with improved renal functions in a phase II study in adult kidney transplant recipients (7). The U.S. Food and Drug Administration (FDA) approved the use of Bela in 2011 based on the findings of two phase III studies, the Belatacept Evaluation of Nephroprotection and Efficacy as First-line Immunosuppression Trial (BENEFIT) and the BENEFIT–Extended Criteria Donors (BENEFIT-EXT) (8, 9). A 3-year outcome analysis of the two studies demonstrated that Bela in combination with mycophenolate (MPA) and low-dose corticosteroids resulted in better renal function and equivalent patient and allograft survival compared to cyclosporine-based regimen, despite an early increased incidence of acute rejections. There was a greater incidence of PTLD in the Bela group after transplantation (10, 11). The 7-year results of the BENEFIT study showed a 43% reduction in the risk of death or graft loss and a significantly higher mean estimated GFR in the Bela groups compared to cyclosporine (12).

In all the above studies (8–12), patients were on steroid immunosuppression. Because of the numerous side effects of corticosteroids (13–17), several transplant centers have instituted protocols that minimize or altogether avoid their usage, especially considering the emergence of new immunosuppressive medications. The safety/efficacy profile of Bela in the steroid-free immunosuppressive regimens has not been evaluated yet.

While both the BENEFIT and BENEFIT-EXT *de novo* studies demonstrated the nephroprotective effects of Bela in kidney transplant recipients, this was associated with increased rates of rejection within the first year post-transplant. Therefore, we aimed to assess the impact of early conversion from tacrolimus to Bela for maintenance immunosuppressive therapy at 3 months post-transplant in adult kidney transplant recipients. We used induction treatment with the lymphocyte depleting agent Alemtuzumab (anti-CD52 antibody) and a steroid free maintenance protocol. We evaluated the effect of this protocol on patient and allograft survival, incidence of rejection and renal graft function. Additionally, the study endpoints included the incidence of adverse events such as new-onset post-transplant diabetes mellitus, hyperlipidemia, infections, and malignancies. The study was designed as a prospective randomized trial with a 24-month follow-up duration.

## Methods

### Study design and patients

In this open-label (not blinded), single center, randomized study (Clinical Trial ID: NCT02213068), kidney allograft recipients received maintenance immunosuppression with tacrolimus (Tac) and mycophenolate (MPA) for the first 3 months post-transplantation and then they were randomized 1:1:1 to undergo conversion to Bela with MPA, Bela with low dose Tac or continue on Tac with MPA. The Institutional Review Board (IRB) of Northwestern University, Chicago, IL, approved the study.

Male and female kidney transplant recipients from living or deceased donors, older than 18 years, EBV seropositive were assessed for eligibility. All subjects needed to demonstrate ability to provide consent and comply with the study procedures. Patients were consented and randomized at 3 months post-transplant.

EBV negative or unknown recipients and those with history of acute rejection within 3 months prior to randomization were excluded from the study. Other exclusion criteria included: pre-transplant donor-specific antibodies, presence of proteinuria >1 g/day or > 0.5 g/day if diabetic in two weeks prior to randomization, rejection on 3-month posttransplant screening biopsy, BK nephropathy on 3-month screening biopsy, positive pregnancy test at the time of randomization, history of previous transplant, history of human immunodeficiency virus (HIV), history of hepatitis C virus, end stage kidney disease (ESKD) secondary to primary focal segmental glomerulosclerosis (FSGS) and participation in an investigational drug or medical device study within 30 days or five drug half-lives, whichever was longer, prior to enrollment into this study.

### Immunosuppression and randomization

All patients received induction with alemtuzumab as a single dose of 30 mg IV over 2 hours intraoperatively and methylprednisolone at 500 mg IV followed with rapid steroid elimination (Methylprednisolone 250 mg post-transplant day 1 and 125 mg post-transplant day 2). All patients were maintained on steroid free regimen with Tac and MPA. MPA was started at the dose of 1000 mg twice/day and adjusted as indicated for leukopenia (WBC<2000/mm^3^). At 3 months post-transplantation, eligible patients who consented to participate in the study were randomized in a 1:1:1 ratio, using a computer-generated list and block randomization, to be converted to Bela with MPA, Bela with low dose Tac or continue with Tac with MPA. Tac was administered to target 12-hour trough level of 8-10 ng/mL during the first 3 months, 7-9 ng/mL from 4-6 months post transplantation and 6-8 ng/mL thereafter. The dose was modified for the second group (Bela with low dose Tac) to maintain a trough level of ≤ 5 ng/mL. Blood Tac trough levels were measured by LC-MS/MS method.

### Outcomes

The primary endpoint of the study was the change in the estimated GFR (eGFR), using MDRD equation, at 2 years posttransplant from the baseline at the time of conversion. The secondary endpoints included patient and renal allograft survival, incidence of biopsy proven acute rejection (BPAR), incidence of *de novo* donor specific antibodies (DSA), and incidence of adverse events such as infections, malignancies, posttransplant diabetes mellitus and hyperlipidemia.

### Covariates

Data collected included immunosuppressive regimen used, recipient demographics such as age, gender, race, case of end-stage kidney disease, history of diabetes mellitus, hypertension, congestive heart failure, need for dialysis prior to transplant, panel reactive antibody level %, degree of HLA mismatch and donor characteristics (living vs. deceased). Posttransplant data included incidence of delayed graft function (DGF), rate of acute rejection, eGFR, patient survival, graft survival, DSA levels, incidence of diabetes, hyperlipidemia (defined as total cholesterol > 200 mg/dL) post-conversion, infections, malignancies and proteinuria.

### Histopathological analyses

To evaluate the impact of this immunosuppressive maintenance regimen on allograft histopathology, we obtained surveillance biopsies at baseline (time of conversion at 3 months post-transplantation), 12 months and 24 months post-transplantation.

### Flow cytometry

We isolated peripheral blood mononuclear cells (PBMC) from peripheral blood by Ficoll gradient and we stored them in liquid nitrogen for batched analysis. For multicolor flow cytometry analyses, we used the following monoclonal antibodies: from Becton Dickinson (BD, Franklin Lakes, NJ), CD3-FITC, CD3-PerCP-Cy5.5, CD4-APC-Cy7, CD8-BV510, CD45RO-FITC, CD45RA-APC, CD45RA-APC-Cy7, CD71-PE, IgD-PerCP-Cy5.5, CD25-APC-Cy7, CD138-BV421, CCR4-PE, CD27-PE, CD95-BV421, CD28-BV421, TIM3-BV421, CCR6-BV421, CCR7-AF700, CXCR3-PE, IL-2-PE, IL-17-BV786, and IFN-γ-PE-Cy7; from Biolegend (San Diego, CA), CD19-BV510, CD56-FITC, CD27-APC, CD127-FITC, CD57-PerCp-Cy5.5, PD-1-APC-Cy7, CXCR5-FITC, LAG3-APC, IL-10-PerCP-Cy5.5, and TNF-α-FITC. From eBioscience (San Diego, CA), CD4-PE-Cy7, CD21-PE, and CD24-APC-Cy7 were used. From Miltenyi Biotec (Auburn, CA), CD25-APC and anti-KLRG1-PE were used. From Beckman Coulter (Indianapolis, IN), CD38-PE-Cy7 was used.

We performed intracellular staining for IL-2, IL-4, IL-17, IFN-g, and TNF-a together with extracellular markers for CD4^+^, CD8^+^, and CD19^+^. Cells were fixed and permeabilized using Intracellular Fixation and Permeabilization Buffer Set (eBioscience) according to the manufacturer’s instructions.

Data were acquired (> 1 × 10^6^ events) on a 3-laser FACSLyric flow cytometer (BD Biosciences) and analyzed with FlowJo^®^ software.

### Gene expression profile

Blood samples collected at 3 months, 12 months, and 24 months post transplantation from a subset of participants underwent next-generation RNA sequencing in the Verici Dx CLIA (Clinical Laboratory Improvement Amendments) laboratory (Franklin, TN) to generate Tuteva risk score results in order to assess the performance of the Verici Dx Tuteva test for the prediction of clinical and subclinical acute rejection (AR).

### Statistical analysis

The baseline data were summarized using descriptive statistics (means and standard deviation for continuous variables and frequency and proportions for the categorical variables). ANOVA test or Wilcoxon rank sum test was used to compare continuous variables. Chi-square or Fisher exact tests was used to compare categorical variables. Patient survival, graft survival, and occurrence of rejections were estimated using Kaplan-Meier survival estimates, where the cumulative estimates of the three groups were compared using the Log-rank test. A mixed-effect model was used to compare the eGFR slopes over time between the three groups.

Flow cytometry results were presented as mean and standard error of the mean. Comparisons of continuous variables between groups was performed by unpaired t-test. Comparisons across serial time-points within the same group was done by ANOVA. P < 0.05 was considered as statistically significant. Statistical analysis was performed using GraphpadPrism^®^ version 9.3.1 software package (Graphpad Software Inc., San Diego, CA) at serial time points.

## Results

### Participants

**Figure 1:**
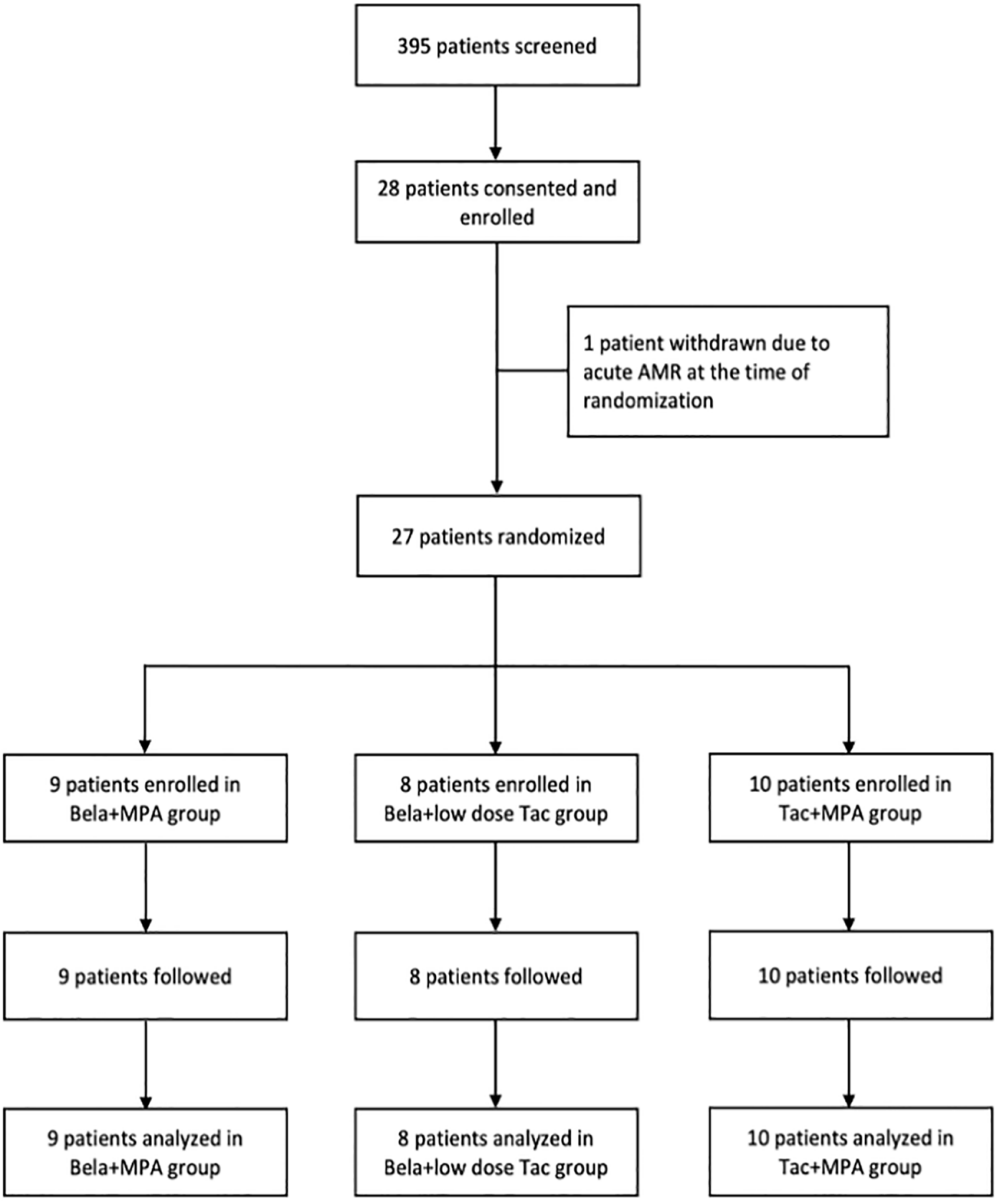
CONSORT diagram. The diagram shows the flow of patients through the study. Twenty seven patients were randomized at 3 months post kidney transplantation to undergo conversion to Bela+MPA (n= 9), Bela+Low dose Tac (n= 8), or continuation of Tac+MPA. The outcomes were analyzed and compared between groups using intention-to-treat (ITT) analysis approach.

Patients were recruited from February 2015 to September 2018. Three hundred and ninety-five patients were screened for eligibility to participate in the study. Twenty-eight patients who met the enrollment criteria consented and were randomized. **Figure 1** illustrates the Consolidated Standards of Reporting Criteria (CONSORT) flow diagram. One participant was withdrawn prior to randomization due to acute antibody mediated rejection at 3-month protocol biopsy. A total of 27 patients were included in the analysis with 9 patients in the Bela+MPA group, 8 patients in the Bela+low dose Tac group and 10 patients in the Tac+MPA group. (**Figure 1**) The baseline characteristics of study participants are summarized in **Table 1**. The three groups were well balanced in terms of age, gender, ethnicity, dialysis need prior to transplant, diabetes mellitus or hypertension, history of coronary artery disease, prior to transplant, history of congestive heart failure, hyperlipidemia and body mass index (BMI). Other characteristics including panel reactive antibody (PRA) titer prior to transplant, degree of HLA mismatch, incidence of delayed graft function (DGF) and baseline mean eGFR were similar in the three groups (**Table 1**).

**Table 1:** Baseline characteristics.

		Bela+MPA	Bela+low dose Tac	MPA+Tac
Characteristic		N= 9	N= 8	N= 10
Age in years (mean (SD))		47.00 (16.21)	49.75 (13.33)	49.70 (13.53)
Male gender (%)		7 (77.78)	3 (37.50)	6 (60.00)
Race (%)	African American	1 (11.11)	3 (37.50)	0 (0.00)
	White	4 (44.44)	3 (37.50)	4 (40.00)
	Hispanic	3 (33.33)	1 (12.50)	5 (50.00)
	Others	1 (11.11)	1 (12.50)	1 (10.00)
Cause of native kidney disease (%)	Polycystic kidney disease	3 (33.33)	1 (12.50)	0 (0.00)
	Diabetes	1 (11.11)	2 (25.00)	3 (30.00)
	Hypertension	3 (33.33)	2 (25.50)	3 (30.00)
	Glomerulonephritis	1 (11.11)	2 (12.50)	2 (20.00)
	Others	1 (11.11)	1 (12.50)	2 (0.00)
Living donors (%)		6 (66.67)	5 (62.50)	3 (30.00)
Preemptive transplant (%)		2 (22.22)	1 (12.50)	3 (30.00)
Years on dialysis (mean (SD))		2.29 (2.53)	2.41 (2.16)	3.59 (4.21)
Diabetes mellitus (%)		0 (0.00)	2 (25.00)	3 (30.00)
Hypertension (%)		8 (88.89)	6 (75.00)	10 (100.00)
Coronary artery disease (%)		0 (0.00)	0 (0.00)	1 (10.00)
Congestive heart failure (%)		0 (0.00)	0 (0.00)	1 (10.00)
Hyperlipidemia (%)		0 (0.00)	0 (0.00)	0 (0.00)
BMI (mean (SD))		24.08 (3.19)	30.48 (5.93)	26.72 (7.05)
HLA mismatch (mean (SD))		2.0(1.5)	2.1(1.6)	2.3(1.3)
FXM Crossmatch	T-lymphocytes	Negative	Negative	Negative
	B-lymphocytes	Negative	Negative	Negative
Panel reactive antibodies %	Class I (median [IQR])	8.00 [4.00, 14.00]	8.00 [0.00, 57.25]	9.50 [0.00, 48.75]
	Class II (median [IQR])	4.00 [4.00,18.00]	5.00[0.00,21.50]	0.00 [0.00,14.00]
DGF (%)		0 (0.00)	0 (0.00)	1 (10.00)
eGFR (mean (SD))		66.33 (17.89)	71.70 (20.70)	67.75 (20.01)

Bela, belatacept; MPA, mycophenolic acid; Tac, tacrolimus; SD, standard deviation; BMI, body mass index; HLA, human leukocyte antigen; IQR, interquartile range; DGF, delayed graft function; eGFR, estimated glomerular filtration rate in ml per minute per 1.73 m^2^. FXM, flow cytometry crossmatch.

### Renal allograft function

**Figure 2:**
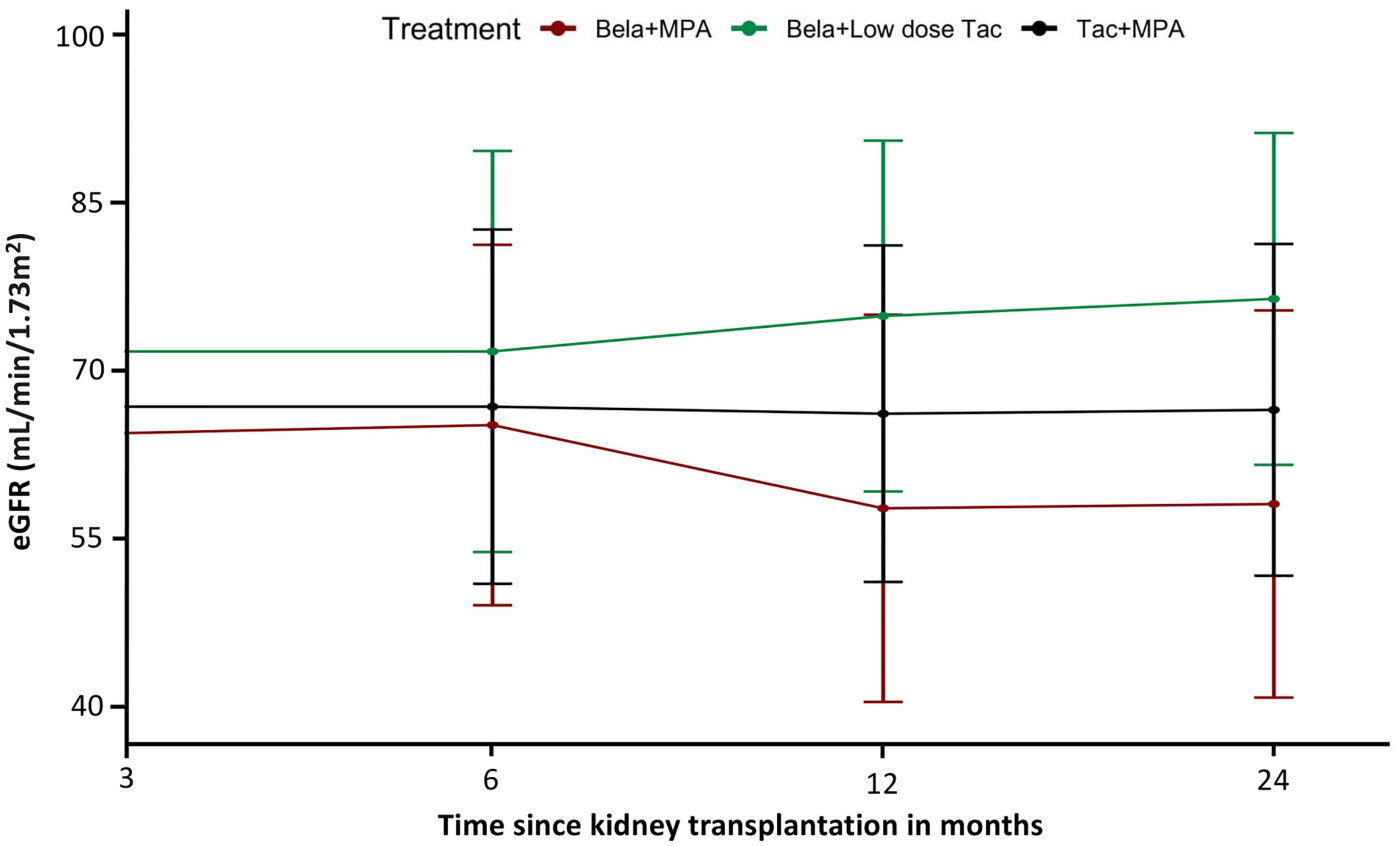
Effect of treatment on estimated glomerular filtration rate (eGFR): Plots of mean eGFR over time from transplantation indicated improvement of eGFR in Bela+Low dose Tac group during the 24-month period. A linear mixed model showed an estimated positive slope of 8.81 mL per minute per 1.73 m^2^ increase in the adjusted eGFR in the Bela+Low dose Tac group and a negative slope of 6.60 mL per minute per 1.73 m^2^ in the Bela+MPA group over 24 months.

The change in the eGFR from the baseline over the two-year follow-up period was -0.38 ml/min/1.73 m^2^ [95% CI, – 4.31 to 0.14] in the Tac+MPA group compared to +8.8 ml/min/1.73 m^2^ [95% CI, 3.81 to 13.81]; P= 0.243, in the Bela+low dose Tac group, and -6.60 ml/min/1.73 m^2^ [95% CI, – 11.33 to 1.87]; P= 497, in the Bela+MPA group (**Figure 2**).

### Patient and renal allograft survival

**Figure 3:**
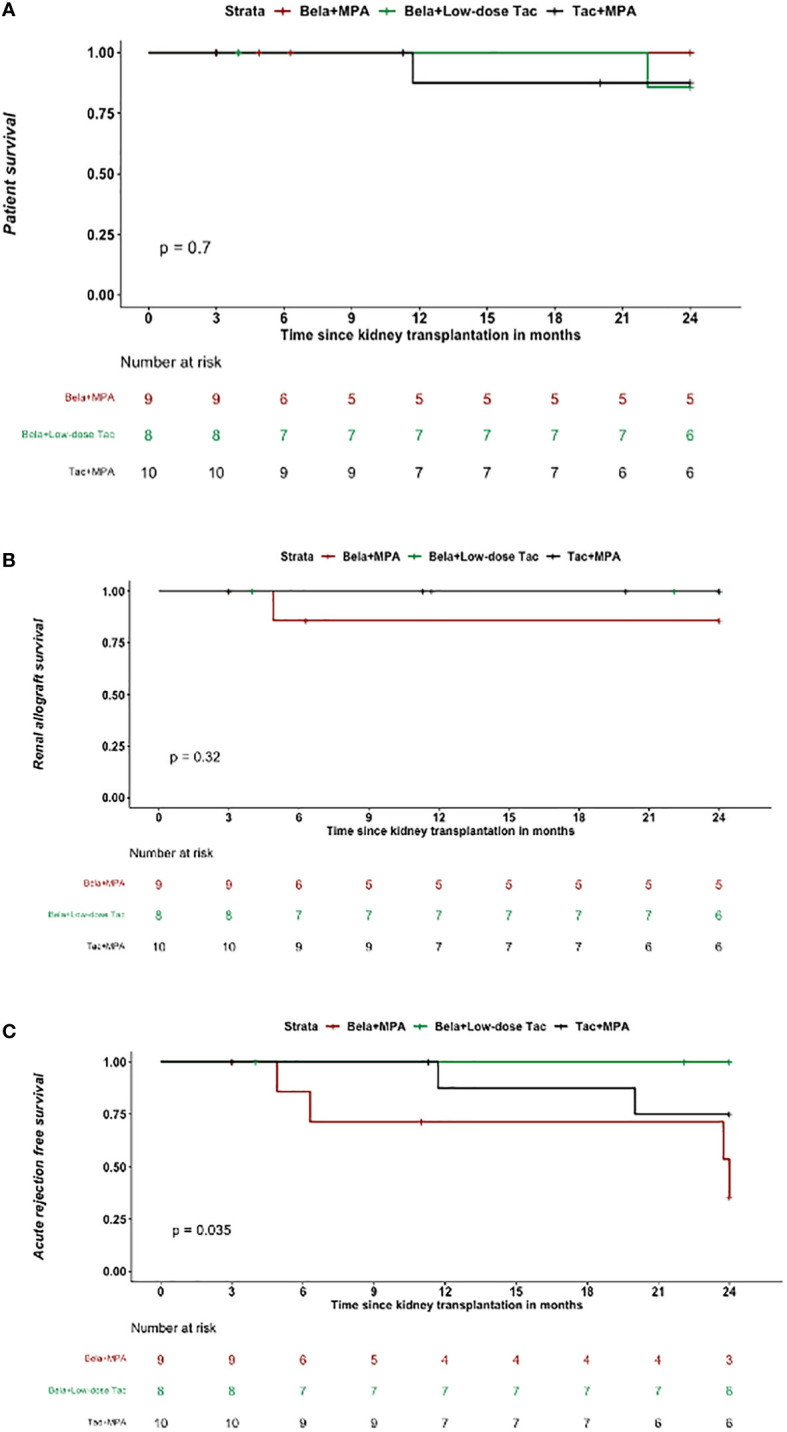
Patient and graft survival and incidence of acute rejection: **(A)** Kaplan-Meier curve depicting the time to occurrence of death in the three treatment groups shows no significant difference of death rate amongst the three treatment groups; **(B)** Kaplan-Meier curve depicting the time to occurrence of renal allograft loss in the three treatment groups shows that there was one graft loss in the Bela+MPA group, and no allograft loss seen in the Bela+Low dose Tac and Tac+MPA groups; **(C)** Kaplan-Meier curve depicting the time to occurrence of BAPR in the three treatment groups shows that Bela+Low dose Tac group was associated with the highest BPAR free survival rate.

Kaplan-Meier patient survival rates were not significantly different across the three groups: the death rates were 0/9 vs. 1/8 vs. 1/10 for Bela+MPA, Bela+low dose Tac, and Tac+MPA groups, respectively (P= 0.7) (**Figure 3A**). The cause of the death in the Bela+low dose Tac group was diabetic ketoacidosis and in the Tac+MPA group death was due to a sepsis secondary to a pneumonia.

The graft survival rates were also similar across the three randomization arms. There was one allograft loss in the Bela+MPA group due to biopsy-proven Banff 2A acute cellular rejection at 5 months posttransplant, which was treated with rabbit antithymocyte globulin and steroids, and the patient was converted back to Tac+MPA. However, the patient remained with significantly impaired kidney function, and was initiated on dialysis at 20 months posttransplant (**Figure 3B**).

### Incidence of acute rejection and DSA

Kaplan-Meier curves of rejection free survival was statistically different across the 3 groups, with lowest rejection free survival in the Bela+MPA group and highest rejection free survival in the Bela+low-dose Tac group (P = 0.035, **Figure 3C**). The rate of biopsy proven acute rejection was not statistically different across the three groups. Even if not statistically different, early conversion to Bela+MPA was associated with a high rate of early acute cellular rejection (4/9), which led to early closure to enrollment to that arm of the study. Except for the case of 2A acute rejection the led to graft loss, the other cases fully recovered graft function upon steroid pulses. At the 24 months post-transplantation, acute cellular rejection was reported in 4/9 patients in the Bela+MPA group, in 0/8 patients in the Bela+low-dose Tac group, and in 2/10 patients in Tac+MPA group (P= 0.087). Antibody mediated rejection was reported in 1/10 patient in the Tac+MPA group, but not observed in Bela+MPA and Bela+low-dose Tac (P= 0.375).

Two patients developed *de novo* DSA in the Tac+MPA group vs. none in the Bela+MPA and Bela+low-dose Tac groups (P= 0.159).

### Safety

There was no significant difference between the three groups in terms of rates of infection, BK viremia, CMV viremia, malignancy, leukopenia, hyperlipidemia and diabetes (**Table 2**).

**Table 2:** Adverse events.

	Bela+MPA	Bela+Low doseTac	Tac+MPA	P value
Adverse event	N= 9	N= 8	N= 10	
CMV viremia (%)	1 (11.11)	1 (12.50)	1 (10.00)	0.986
BK viremia (%)	3 (33.33)	4 (50.00)	1 (10.00)	0.163
Other infections (%) ^*^	1 (11.11)	4 (50.00)	2 (20.00)	0.174
Leukopenia (%)	3 (33.33)	3 (37.50)	2 (20.00)	0.690
De novo DSA (%)	0 (0.00)	0 (0.00)	1 (20.00)	0.159
Hyperlipidemia (%)	1 (11.11)	0 (0.00)	3 (30.00)	0.190
New onset DM (%)	0 (00.00)	0 (00.00)	1 (10.00)	0.414
Malignancy (%)	0 (00.00)	0 (00.00)	1 (10.00) ^†^	0.414

^*^Other infections included herpes-zoster, blastomycosis, pneumonia and Clostridium difficile.

^†^ One patient developed skin squamous cell carcinoma

### Graft histopathology

We analyzed the histopathological data of patients who completed 24-month follow up period. There was no significant difference in interstitial fibrosis/tubular atrophy, glomerulosclerosis, arteriosclerosis, arteriolar hyalinosis and transplant glomerulopathy between the three groups at 3-, 12-, or, 24-month surveillance biopsies.

### Immune phenotype

We performed extensive immune phenotyping of PBMC collected from all the participants at 3 months (time of randomization), and at 6, 12, and 24 months posttransplant. At 3 months, none of the T cell subsets (including naive, memory, and Treg) were fully recovered after alemtuzumab depleting induction, but they did not significantly change across the three treatment groups during the follow-up period (**Supplemental Figures 1** **–** **3**). Trends in B cell subsets did also not significantly differ across the three groups.

**Figure 4:**
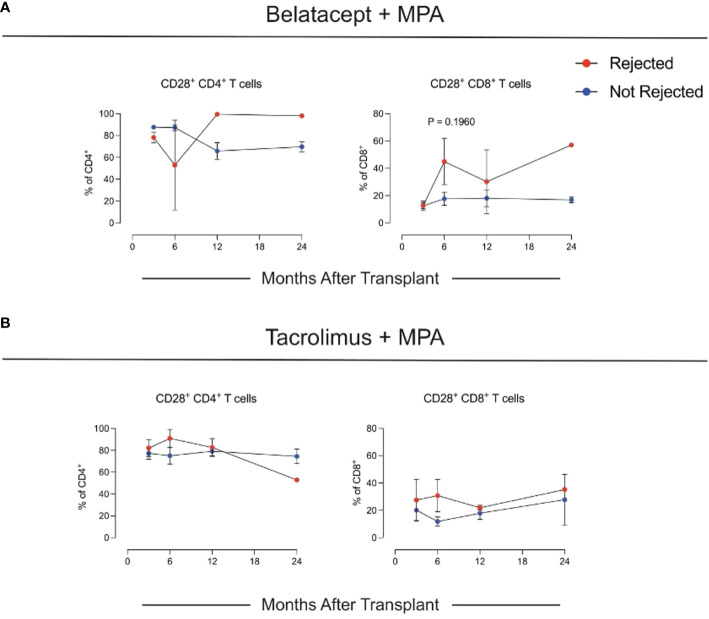
Changes in CD28^+^ T cells. **(A)** Changes in CD28+ CD4+ and CD8+ T cells in the Belatacept + MPA and **(B)** in the Tacrolimus + MPA cohorts.

CD28^+^ T cells have been associated with an increased risk of acute rejection in patients on Bela, but not on Tac, suggesting that these cells are resistant to Bela and may be implicated in the acute rejection episodes occurring after conversion from Tac (18). In our study, CD28^+^CD4^+^ and CD28^+^CD8^+^ T cells were higher in Bela + MPA patients with acute rejection compared to patients without rejection, although the difference did not meet statistical significance (**Figure 4**).

### Gene expression profile

The Tuteva test is a lab-developed test (LDT) that is performed centrally in the Verici Dx laboratory in accordance with CLIA regulations. The Tuteva test AR risk score results of 21 participants demonstrated a negative predictive value of 91.67% [95% CI, 63.28% to 98.60%], a positive predictive value of 70% [95% CI, 45.28% to 86.81%], a sensitivity of 87.50% [95% CI, 47.35% to 99.68%], a specificity of 78.57% [95% CI, 49.20% to 95.34%], an accuracy of 81.82% [95%ci CI, 59.72% to 94.81%], and an area under the receiver operating characteristic curve of 0.83 for predicting biopsy proven borderline rejection or acute renal allograft rejection. The prevalence of borderline changes/acute rejection in this group of participants was 36.36%.

## Discussion

Calcineurin inhibitor avoidance in kidney transplantation is challenging and has been associated with high rejection risk. Since its approval in 2011, Bela has allowed CNIA and resulted in reduced nephrotoxicity and *de novo* DSA formation despite an early increase in acute rejections.

Our study of steroid-free kidney transplant recipients indicates that early conversion from Tac to Bela in combination with MPA is associated with a high rate of early acute cellular rejection (4/9), which led to early closure to enrollment to that arm of the study. On the other hand, our data showed that conversion to Bela + low-dose Tac (targeting a goal tacrolimus trough level ≤5 ng/mL) is an effective steroid-free maintenance immunosuppressive regimen and was associated with a +8.81 ml per minute per 1.73 m^2^ increase in the eGFR at 24 months post-transplantation. There were no early acute rejections, renal graft loss or development of *de novo* DSA in patients randomized to that treatment arm. Our findings confirm and expand the findings form the BENEFIT and BENEFIT-EXT trials. In contrast to those two studies, our study used 1) the lymphocyte depleting agent Alemtuzumab for induction along with early steroid withdrawal; 2) Tac, rather than CSA, which was used in BENEFIT and BENEFIT-EXT; and 3) our study included patients with high PRA, which were excluded in those trials.

In the Bela+MPA group there was one renal allograft loss, which was associated with early Banff 2A acute cellular rejection. Despite early treatment with anti-thymocyte globulin, steroids, and conversion to Tac+MPA, the allograft function did not recover, and the patient was initiated on hemodialysis 20 months post transplantation. The high rate of rejections probably impacted also the eGFR that was numerically lower in the Bela+MPA group at the end of the study.

Our extensive immune phenotyping analysis of PBMCs did not reveal significant differences across groups. Intriguingly, we found that patients who experienced rejection in the Bela + MPA group had numerically higher levels of CD28^+^ T cells in the circulation. This is consistent with the hypothesis that CD28^+^ effector memory T cells, capable of producing high levels of IL-2, are a biomarker for rejection risk in Bela patients (17, 19–21)

In the subset of participants who underwent peripheral blood transcriptomic profiling, we found that the Verici Dx Tuteva clinical and sub-clinical acute rejection risk score test had a high negative predictive value (91.67%) and a fair positive predictive value (70%) for predicting biopsy proven borderline changes/acute rejection. Further study of this newly available test in a larger cohort of patients is needed.

Belatacept was well tolerated by our cohort, and there were no significant differences across the three groups in the incidence rates of infection, malignancy, neutropenia, hyperlipidemia, or diabetes, further supporting the safety of Bela regimens that include T-cell depletion induction therapy. There were no PTLD cases in our study of EBV seropositive patients, supporting the concept that PTLD does not represent a safety concern in EBV seropositive patients.

There are a few caveats to consider when interpreting our study findings. First, the small sample size limited the power of our study and generalizability of our findings. The relatively short follow-up also reduced our chances to detect as statistically significant the different trends in eGFR across treatment arms, although serial surveillance graft biopsies did not reveal major differences. Due to the limited availability of Alemtuzumab in many countries, generalizability of our findings might be limited.

In conclusion, our study evaluated the impact of early conversion of CNIs at 3 months to Bela in a steroid-free maintenance immunosuppressive therapy following induction with Alemtuzumab. Our findings indicate that early conversion to Bela helps reduce the required maintenance dose of CNI to a lower level that potentially improves renal allograft function without the development of early rejections. The findings also suggest that early conversion to Bela in combination with mycophenolate without steroids is associated with a high rate of early rejection and potentially lower renal allograft function or even allograft loss. Further evaluation of a Bela-based maintenance regimen in a larger cohort of kidney transplant recipients using a steroid-free regimen is needed to support these findings.

## Data availability statement

The raw data supporting the conclusions of this article will be made available by the authors, without undue reservation.

## Ethics statement

The studies involving human participants were reviewed and approved by the Institutional Review Board (IRB) of Northwestern University, Chicago, IL. The patients/participants provided their written informed consent to participate in this study.

## Author contributions

IT participated in the research design, data analysis and writing the paper. PH, FY, MS-B and AI participated in the writing of the paper. JL and MA participated in the performance of the research. PC and SB participated by immune phenotyping and proofreading the manuscript draft. LG was the principal investigator and the supervisor for this study. All authors contributed to the article and approved the submitted version.
