# Supplementary figures and images for: Early calcineurin-inhibitor to belatacept conversion in steroid-free kidney transplant recipients
Source: Front Immunol. 2022 Dec 19;13:1096881. doi: 10.3389/fimmu.2022.1096881 (PMC9806416; doi:10.3389/fimmu.2022.1096881)

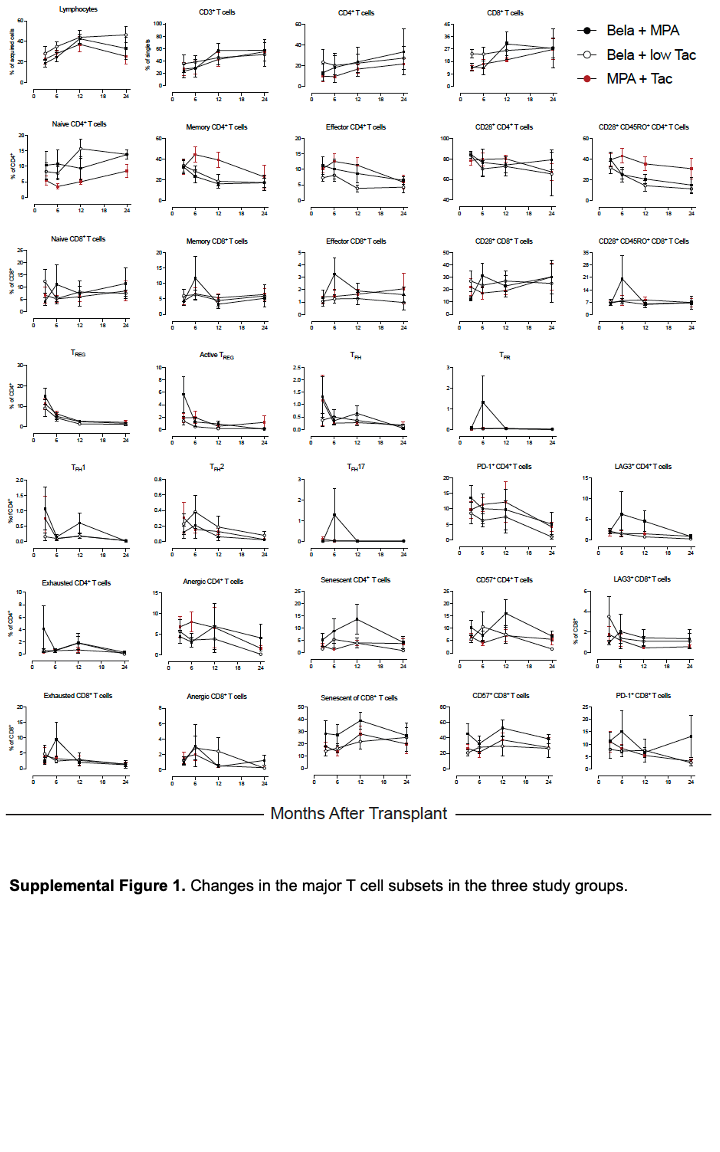

Supplement: Supplementary file 1 [file Image_1.tiff]

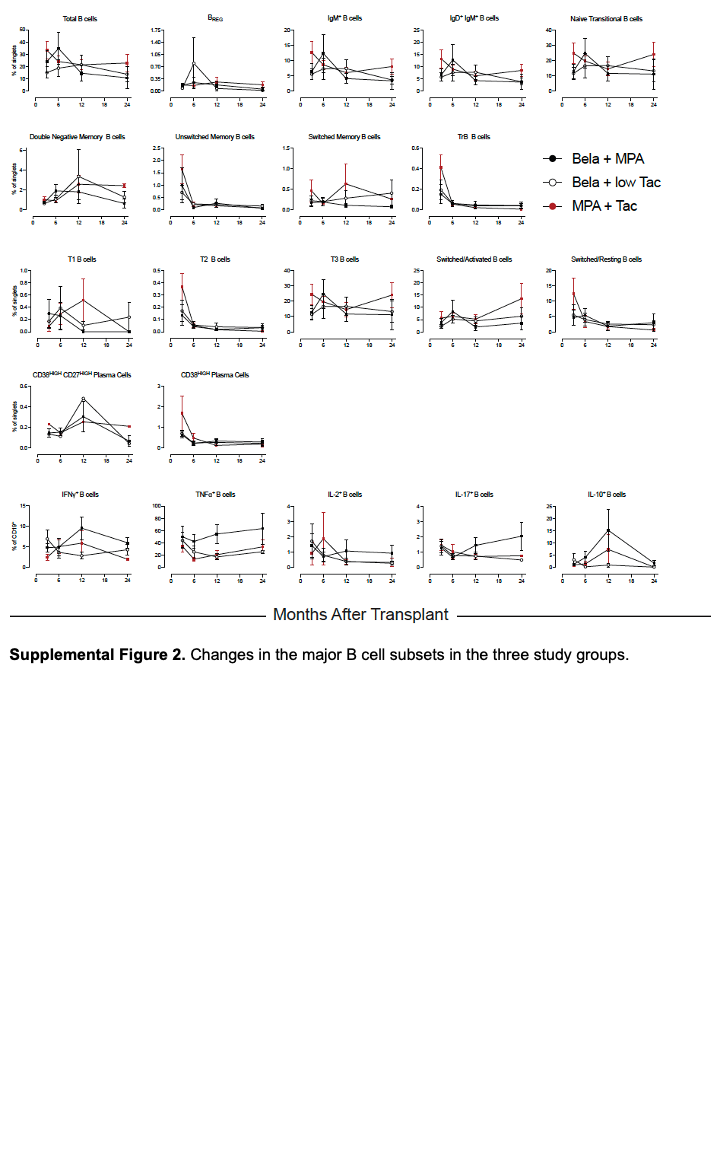

Supplement: Supplementary file 2 [file Image_2.tiff]

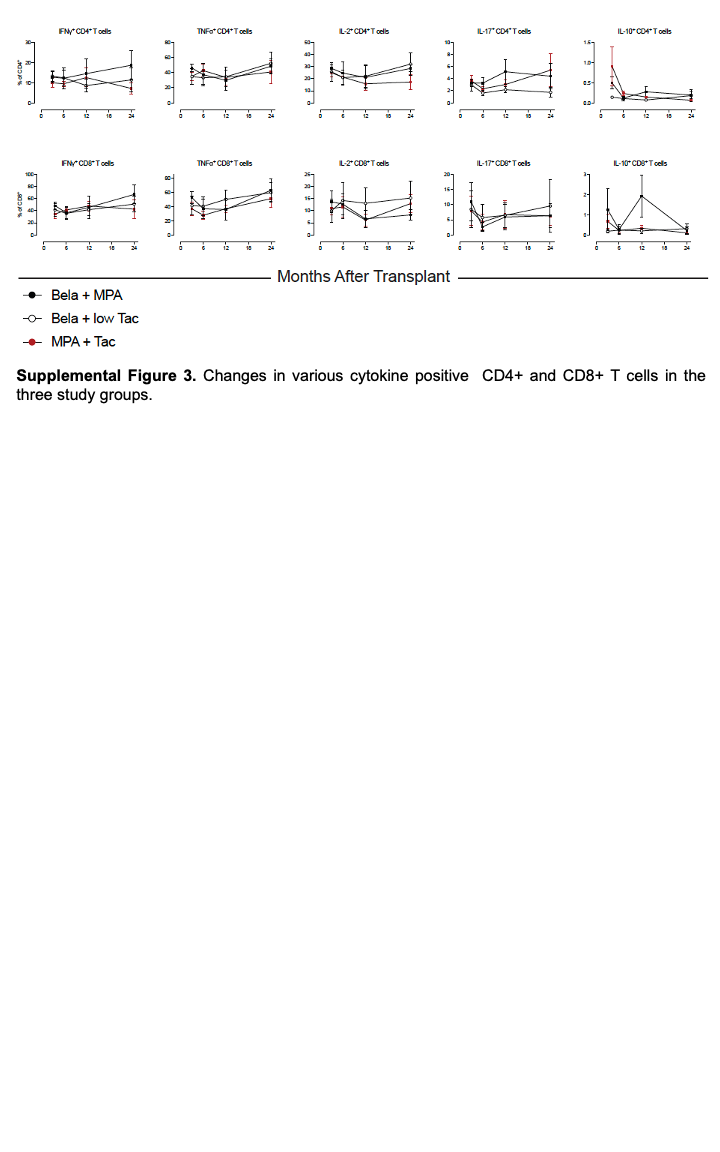

Supplement: Supplementary file 3 [file Image_3.tiff]
